# Development of a virtual phantom for HDR cervical cancer brachytherapy: A multi‐institutional tool for training, quality assurance, and variability reduction

**DOI:** 10.1002/acm2.70620

**Published:** 2026-06-07

**Authors:** Kotaro Iijima, Hiroyuki Okamoto, Takahiro Ushijima, Yoshihiro Ueda, Yoshifumi Oku, Masahiko Toyota, Yasushi Ono, Jun Takatsu, Jun‐ichi Fukunaga, Naoya Murakami, Tatsuya Ohno

**Affiliations:** ^1^ Department of Radiation Oncology Graduate School of Medicine, Juntendo University Bunkyo‐ku Tokyo Japan; ^2^ Section of Radiation Safety and Quality Assurance Division National Cancer Center Hospital Chuo‐ku Tokyo Japan; ^3^ Department of Radiology Mie University Hospital Tsu Mie Japan; ^4^ Department of Radiation Oncology Osaka International Cancer Institute Chuo‐ku Osaka Japan; ^5^ Division of Radiology, Department of Clinical Technology Kagoshima University Hospital Kagoshima‐City Kagoshima Japan; ^6^ Division of Radiology, Department of Clinical Technology Tottori University Hospital Yonago‐City Tottori Japan; ^7^ Department of Radiation Oncology, Faculty of Medicine Juntendo University Bunkyo‐ku Tokyo Japan; ^8^ Division of Radiology, Department of Medical Technology Kyushu University Hospital Higashi‐ ku Fukuoka Japan; ^9^ Department of Radiation Oncology Gunma University Graduate School of Medicine Maebashi Gunma Japan

**Keywords:** brachytherapy, development, virtual phantom

## Abstract

**Background:**

High‐dose‐rate brachytherapy (HDR‐BT) is a critical modality in the treatment of cervical cancer, requiring accurate and efficient treatment planning. Unlike external beam radiation therapy, HDR‐BT demands specialized skills and training. However, accessible and standardized educational tools—particularly virtual phantoms—remain limited.

**Purpose:**

This study aimed to develop a virtual phantom tailored for HDR‐BT treatment planning in cervical cancer management. The objective was to support planner training, reduce interobserver error, and provide a scalable platform for quality assurance and educational use.

**Methods:**

Four virtual phantoms (VP1–VP4) were generated using computed tomography images of physical models equipped with different applicators (Fletcher, Geneva, VCMC, and Venezia). Dose constraints were initially established through a single‐institutional planning study (SIPS) and validated via a multi‐institutional planning study involving seven institutions. Dose–volume parameters (DVPs) were analyzed to assess compliance with the constraints and to quantify inter‐planner error (IPE) and inter‐institutional error (IIE). The coefficient of variation (CV) and F‐tests were employed to evaluate variability.

**Results:**

Initial compliance with SIPS‐defined dose constraints ranged from 35.4% to 70.8%. As compliance was low across all phantoms, we adjusted the spatial relationships between the target volume and organs at risk. The compliance then improved to 83.3%‐100.0%. IIE was greater than IPE, with several DVPs exhibiting CVs above 20%. The hyper‐dose sleeve (V200%) demonstrated the highest variability, with CVs exceeding 40% in some cases. These findings emphasize the importance of standardized training tools for reducing interobserver variability.

**Conclusions:**

The developed virtual phantoms offer practical and accessible tools for HDR‐BT treatment planning education. Its digital format enables broad dissemination and adaptability across institutions, supporting both training and clinical use. Ongoing refinement will be necessary to ensure continued relevance in evolving clinical environments and contribution to consistent, high‐quality care in gynecologic brachytherapy.

## 1 INTRODUCTION

High‐dose‐rate brachytherapy (HDR‐BT) is an advanced radiation therapy technique that requires surgical maneuvers; procedural management; and rapid, accurate treatment planning. These skills differ markedly from those required for external beam radiation therapy (EBRT) and therefore call for dedicated training approaches. In response, several training systems and educational workshops have been developed and implemented.^1^, ^2^, ^3^, ^4^, ^5^, ^6^, ^7^, ^8^, ^9^, ^10^

Broadly, HDR‐BT training can be categorized into two primary domains: surgical maneuvers and treatment planning. While surgical training typically requires dedicated physical phantoms and must be conducted on‐site—thereby limiting accessibility—treatment planning can be carried out remotely, provided that suitable planning systems and educational tools are available.

For HDR‐BT treatment planning to be effective, three key attributes are essential: speed, to alleviate patient burden and minimize intrafractional displacement of applicators and needles^10^, ^11^; accuracy, to prevent near‐miss incidents such as errors in applicator reconstruction or dose prescription^12^; and optimal dose distribution, to ensure therapeutic efficacy.^13^ Mastering these elements is essential for safe and effective clinical practice, yet doing so remains considerably challenging—particularly for novice practitioners.

Accordingly, developing accessible and effective training tools for treatment planning is vital to advancing HDR‐BT. However, most existing tools rely on specialized physical phantoms that are not readily available at many institutions. Although facilities with established HDR‐BT programs may train personnel using historical clinical cases, centers newly adopting the technique often lack such resources. This disparity underscores the need for flexible, scalable, and broadly accessible training platforms that can support the safe implementation of HDR‐BT across diverse clinical settings.

Unlike EBRT, where virtual phantoms are widely employed for treatment planning training, commissioning, and quality assurance/quality control (QA/QC),^14^ no standardized virtual phantom currently exists for HDR‐BT. For instance, American Association of Physicists in Medicine Task Group 119 (TG‐119) offers four virtual phantoms of varying complexities; these have been extensively used for both educational and QA/QC purposes in EBRT.^15^, ^16^, ^17^, ^18^, ^19^, ^20^ The lack of similar standardized tools in HDR‐BT has compelled individual institutions to develop their own approaches for treatment planning training and system QA/QC,^20^, ^21^, ^22^, ^23^, ^24^, ^25^ resulting in variability across clinical settings.

To address this gap, we developed a virtual phantom specifically designed for HDR‐BT treatment planning. This report outlines the phantom's development methodology and structure, explains the rationale and process used to define dose constraints, and describes its intended applications in both clinical and educational contexts. We also present findings from a multi‐institutional planning study (MIPS) conducted to: (i) evaluate the validity of dose constraints, (ii) assess inter‐institutional variability in treatment planning, and (iii) demonstrate the broader applicability and standardization potential of this tool. Notably, this study was initiated by a Japanese working group convened to investigate dose optimization in HDR‐BT.

## 2 METHODS

### 2.1 Virtual phantom fabrication

**FIGURE 1:**
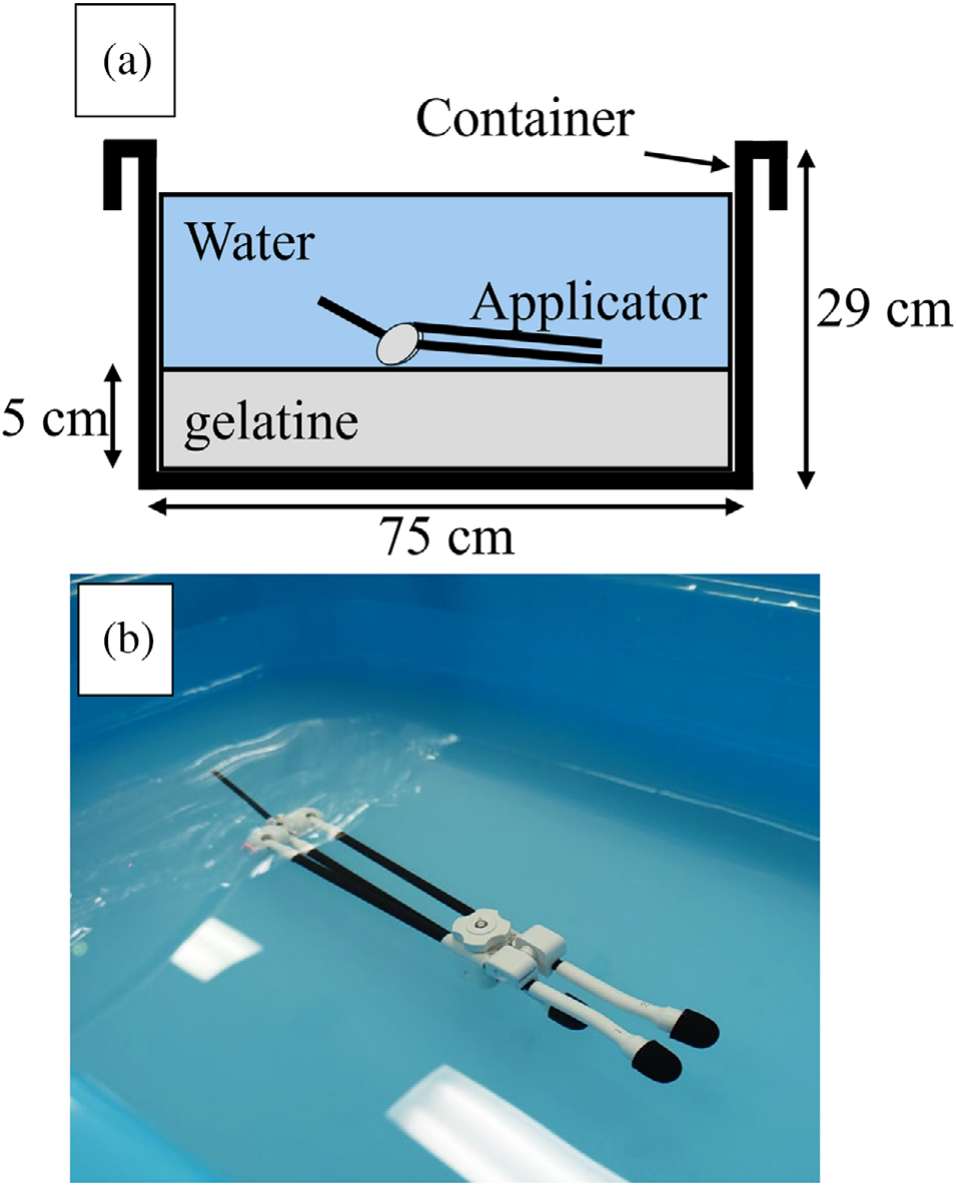
Overview of the physical phantom used in the development of the virtual phantom. The phantom consisted of a container measuring 53 cm in width, 75 cm in depth, and 29 cm in height. A 5‐cm‐thick gelatin layer was placed at the bottom to provide structural support, and applicators were embedded into it. The container was then filled with water to simulate the surrounding tissue environment. (A) Overview of the physical phantom. (B) Photograph of the physical phantom.

A physical phantom was first fabricated to serve as the structural basis for the virtual phantom. Initially, at the bottom of a water tank measuring 53 cm in width, 75 cm in depth, and 29 cm in height, a 5‐cm‐thick gelatin base was prepared. The tank was then filled with water (Figure 1), and applicators were embedded into the gelatin base. Computed tomography (CT) images of four virtual phantom configurations were subsequently acquired using the Aquilion ONE scanner (Canon Medical Systems, Otawara, Japan) under the helical scan mode at a tube voltage of 120 kVp, tube current of 484 mA, and slice thickness of 2 mm. Images were reconstructed using filtered back projection with a standard kernel and a matrix size of 512 × 512. The field of view was set to 500 mm. Four virtual phantoms, each representing a different applicator configuration, were generated: (1) a Fletcher applicator (VP1); (2) a Geneva applicator with three ProGuide sharp needles (VP2); (3) a vaginal CT/MR multi‐channel (VCMC) applicator with three ProGuide round needles (VP3); and (4) a Venezia applicator with three ProGuide sharp needles (VP4). VP1 is designed without needles to replicate the most common form of gynecologic intracavitary brachytherapy. Applicators VP2 to VP4 were equipped with interstitial needle guides and the needle positions were determined based on clinical experience to construct the virtual phantoms. The obtained images were transferred to MIM Maestro version 7.3.5 (MIM Software Inc., Cleveland, OH, USA). Using MIM's contouring tools, a medical physicist (10 years of experience) and a physician (18 years of experience) jointly delineated the high‐risk clinical target volume (CTV_HR_), bladder, rectum, sigmoid colon, and small bowel for each applicator. The physician contoured the CTV_HR_ based on multiple clinical images, considering the placement of the applicator and needles. The rectum was delineated as a cylindrical structure, the bowel as an elliptical structure, the duodenum as a structure connecting the rectum and bowel, and the bladder as a rectangular structure. These contours were positioned with reference to the distances between the CTV_HR_ and each OAR observed in the clinical images. The phantom was developed solely for treatment planning systems involving TG43–based dose calculations.^27^

The applicators used in this study were loaned by Chiyoda Technol Corporation (Tokyo, Japan).

### 2.2 Dose constraint determination

The virtual phantoms were also designed to support treatment planning practice in HDR‐BT. To accommodate varying levels of expertise, two planning levels were introduced: an easy version and a hard version. The easy version was defined as the minimum constraints that must be achieved. As clinical practice often requires more stringent constraints than the EMBRACE II constraints, we also established the hard version constraints. The dose constraints for the easy version were based on previous reports,^28^, ^29^, ^30^, ^31^, ^32^, ^33^, ^34^, ^35^, ^36^, ^37^ assuming the uniform delivery of 45 Gy in 25 fractions via EBRT, followed by four HDR‐BT sessions of 6 Gy each. Under this assumption, the cumulative CTV_HR_ D_90%_, expressed as equivalent dose in 2 Gy fractions (EQD2), was intended to exceed either 80 or 85 Gy. The constraints listed in Table 1 represent the required physical dose per HDR‐BT session to meet this goal. The hard version constraints were established through a single‐institutional planning study (SIPS) conducted at the facility where the virtual phantoms were primarily developed. During this study, six medical physicists, each with three or more years of experience in brachytherapy, generated treatment plans for VP1–VP4 using Oncentra Brachy (Elekta) under the easy‐version dose constraints presented in Table 1. Treatment plans using ^192^Ir in which the reconstruction, offset, and indexer settings had been completed (Table 2) but in which the dwell positions and dwell times had not yet been entered, were provided to six participants. The participants were then requested to generate dose distributions. As inverse planning is not used for gynecologic HDR brachytherapy at our institution, it was not employed in this study. Six medical physicists were included in the SIPS to determine the hard version dose constraints and to assess inter‐planner error (IPE) within a single institution. Moreover, seven institutions were included in the MIPS to: (i) validate the dose constraints established in the SIPS and (ii) evaluate inter‐institutional error (IIE). The number of participating institutions was determined by availability to ensure that the results were more reliable and less susceptible to bias from a single institution. The planners were provided only with applicator details—specifically, reconstruction numbers, indexer lengths, and offset values—and were instructed to maximize CTV_HR_ dose coverage while minimizing dose to the OARs. Beyond these instructions, they were free to develop treatment plans at their own discretion. From the resulting plans, dose–volume parameters (DVPs) were extracted, including V_98%_, V_100%_, V_200%_, and D_90%_ for the CTV_HR_, as well as D_2cc_ values for the bladder, rectum, sigmoid colon, and small bowel. These DVPs were analyzed using Microsoft Excel, and the resulting mean values were adopted as the dose constraints for the hard version.

**TABLE 1:** Dose constraints for the easy version of the virtual phantoms. These constraints are uniformly applied to all virtual phantoms (VP1–VP4). Following uniform irradiation of 45 Gy in 25 fractions that had been uniformly delivered via EBRT, an HDR‐BT schedule of four sessions (6 Gy each) was assumed.

CTV_HR_ D_90%_ [Gy]	Rectum D_2cm3_ [Gy]	Bladder D_2cm3_ [Gy]	Sigmoid D_2cm3_ [Gy]	Bowel D_2cm3_ [Gy]
7.1	4.5	5.5	4.5	4.5

These constraints are uniformly applied to all virtual phantoms (VP1–VP4), assuming uniform irradiation of 45 Gy in 25 fractions delivered via EBRT, followed by an HDR‐BT schedule of four sessions (6 Gy each).

**TABLE 2:** The offset, indexer and prescribed dose of the virtual phantoms. VP1: Fletcher applicator; VP2: Geneva applicator; VP3: VCMC applicator; VP4: Venezia applicator.

	Applicator	Offset [mm]	Indexer [mm]	Prescribed Dose [Gy]
VP1	Tandem	−6	microSelectron: 1500	6
			Flexitron: 1300	
	Ovoid	−5	microSelectron: 1500	
			Flexitron: 1300	
VP2	Tandem	−6	microSelectron: 1500	6
			Flexitron: 1300	
	Ovoid	−6	microSelectron: 1500	
			Flexitron: 1300	
	ProGuide sharp needle	−4	microSelectron: 1240	
			Flexitron: 1234	
VP3	Tandem	−5	microSelectron: 1500	6
			Flexitron: 1300	
	ProGuide round needle	−4	microSelectron: 1293	
			Flexitron: 1288	
VP4	Tandem	−5	microSelectron: 1500	6
			Flexitron: 1300	
	Ovoid	−5	microSelectron: 1500	
			Flexitron: 1300	
	ProGuide sharp needle	−4	microSelectron: 1240	
			Flexitron: 1234	

VP1: Fletcher applicator; VP2: Geneva applicator; VP3: VCMC applicator; VP4: Venezia applicator.

### 2.3 Validation of dose constraints

The dose constraints established in the SIPS were validated through a MIPS conducted across seven institutions. All planners held at least eight years of clinical experience. During this study, one medical physicist or radiation technologist from each institution generated treatment plans for VP1 through VP4. Each planner was instructed to perform the treatment plan using Oncentra Brachy and to meet the SIPS‐established dose constraints during plan development (Table 1) using the specified applicator information—namely, offset values, indexer lengths, and catheter numbers (Table 2)—while all other planning parameters were left to their discretion. The resulting plans were imported into Oncentra Brachy, and DVPs were extracted, including D_90%_, D_98%_, V_100%_, and V_200%_ for the CTV_HR_, along with D_2cc_ values for the OARs. For any virtual phantom with a dose constraint compliance rate below 80%, planning difficulty was adjusted by modifying the spatial relationship between the CTV_HR_ and OARs using the contour manipulation tools in MIM Maestro. During this process, the OAR positions were simply geometrically translated to increase their distance from the CTVHR, which was fixed. Geometric translation were based on the MIPS plan that achieved the highest CTV_HR_ D_90%_ and D_98%_. This approach ensured that the virtual phantoms could support treatment plans satisfying both CTV_HR_ and OAR dose constraints. Further, to evaluate the IPE in the SIPS and the IIE in the MIPS, DVPs from both studies were analyzed. The coefficient of variation (CV) was calculated based on the mean and standard deviation of the DVPs for the CTV_HR_ and OARs. Subsequently, based on the F‐value derived from the CV, an F‐test was conducted to assess whether the variation between the IPE and IIE was statistically significant. A 5% significance level was used. The *F*‐test was conducted using Excel's built‐in statistical functions.

## 3 RESULTS

### 3.1 Virtual phantoms

**FIGURE 2:**
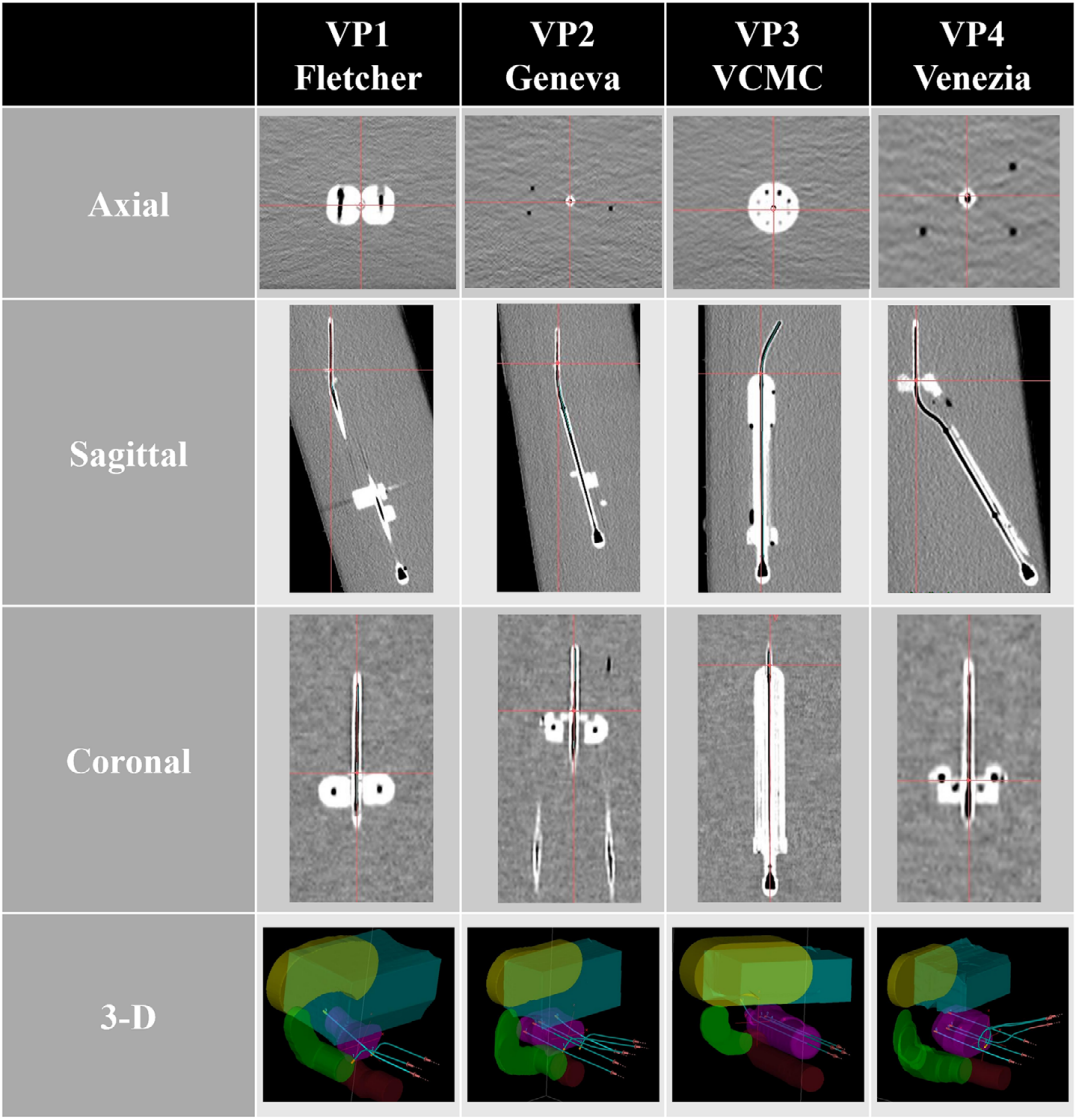
Configurations of the four virtual phantoms. VP1: Fletcher CT/MR applicator; VP2: Geneva applicator with three ProGuide sharp needles; VP3: vaginal CT/MR multi‐channel applicator with three ProGuide round needles; VP4: Venezia applicator with three ProGuide sharp needles. The 3D views depict the spatial arrangement of anatomical structures and applicators. Yellow indicates the bowel bag, green the sigmoid colon, brown the rectum, blue the bladder, and pink the high‐risk clinical target volume.

The developed virtual phantoms are presented in Figure 2. VP1 through VP4 correspond to phantoms incorporating the Fletcher, Geneva (with three ProGuide sharp needles), VCMC (with three ProGuide round needles), and Venezia (with three ProGuide sharp needles) applicators, respectively.

### 3.2 Dose constraint determination

**FIGURE 3:**
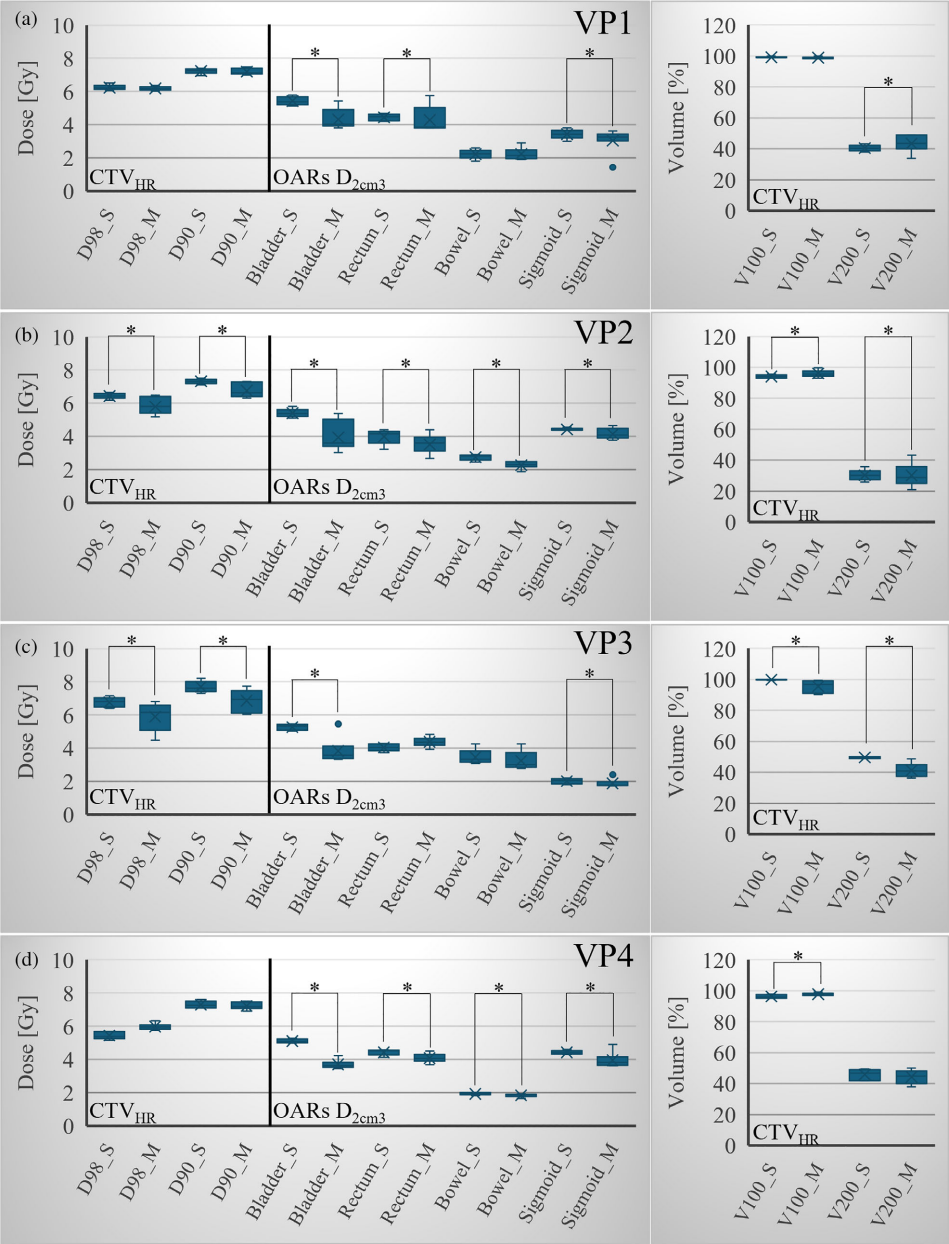
Box plots of dose–volume parameters (DVPs) for each virtual phantom in the single‐institutional planning study (SIPS) and the multi‐institutional planning study (MIPS). “S” and “M” on the horizontal axis represent the SIPS and MIPS, respectively. A, B, C, and D correspond to the Fletcher applicator (VP1), Geneva applicator (VP2), VCMC applicator (VP3), and Venezia applicator (VP4), respectively. * indicates a DVP for which a significant difference was obtained by the F‐test.

Figure 3 shows the SIPS results. The different CTV_HR_ coverages of the VP1 and VP2 phantoms, both constructed using a tandem‑and‑ovoid applicator, is likely attributable to the applicator design. Particularly, as VP2 is designed for larger tumors requiring needle insertion than VP1, treatment planning is more challenging for VP2 than for VP1. The mean values obtained from these results were adopted as the dose constraints for the virtual phantoms and are summarized in Table 3.

**TABLE 3:** Dose constraints for the hard version of the virtual phantoms, established based on the results of the single‐institutional planning study. VP1: Fletcher applicator; VP2: Geneva applicator; VP3: VCMC applicator; VP4: Venezia applicator.

No. of virtual phantoms	VP1	VP2	VP3	VP4
CTV_HR_ D_98%_ [Gy]	6.2	5.4	6.8	5.4
CTV_HR_ D_90%_ [Gy]	7.2	7.3	7.7	7.3
CTV_HR_ V_100%_ [%]	99	94	100	96
CTV_HR_ V_200%_ [%]	40	30	50	46
Rectum D_2cm3_ [Gy]	4.4	4.0	4.0	4.4
Bladder D_2cm3_ [Gy]	5.4	5.4	5.2	5.1
Sigmoid D_2cm3_ [Gy]	3.4	4.4	2.0	4.4
Bowel D_2cm3_ [Gy]	2.2	2.7	3.5	1.9

VP1: Fletcher applicator; VP2: Geneva applicator; VP3: VCMC applicator; VP4: Venezia applicator. The planning objective was to achieve D_98%_, D_90%_, and V_100%_, while keeping V_200%_ and D_2cm3_ below their respective limits.

### 3.3 Validation of dose constraints

**FIGURE 4:**
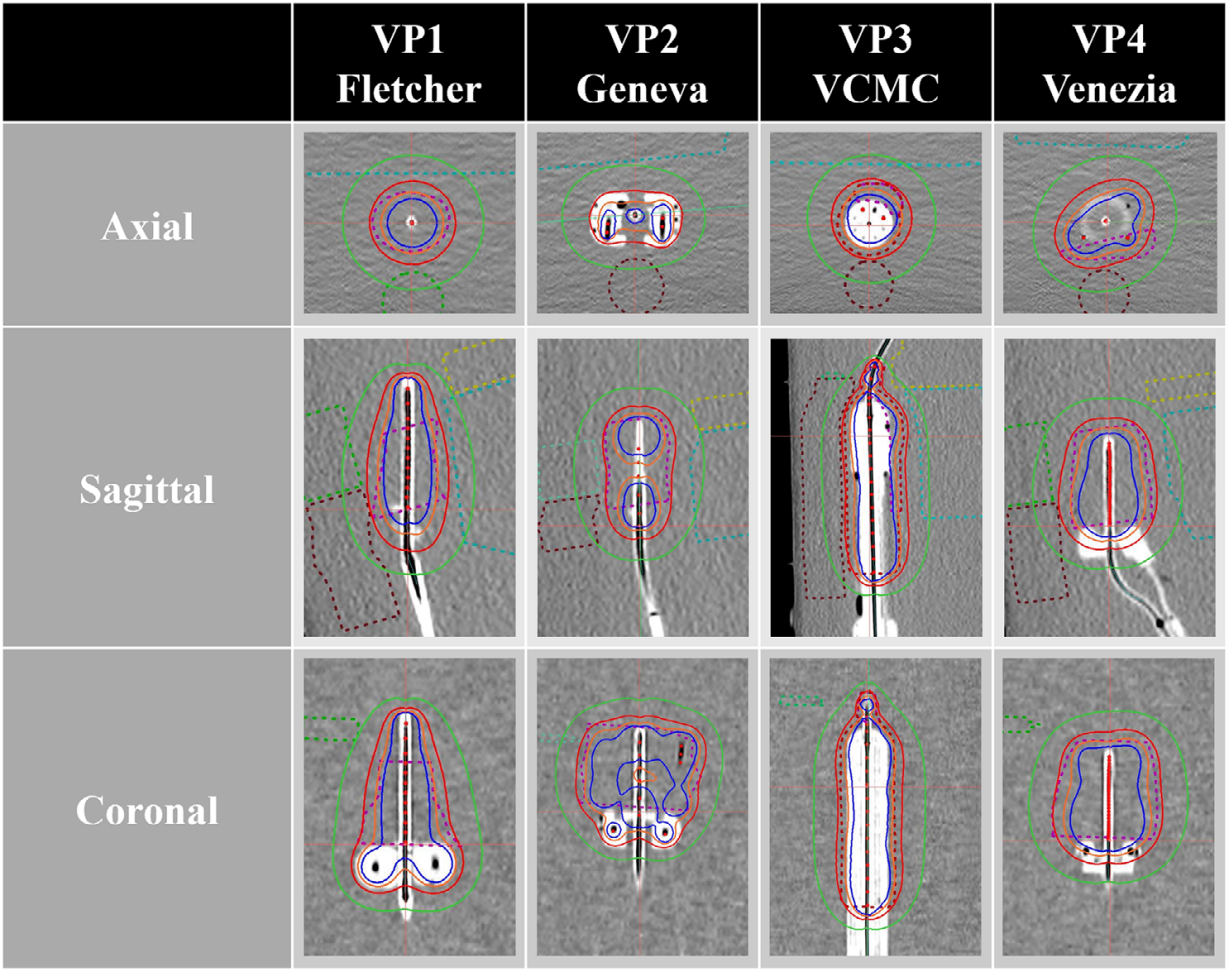
Dose distributions for each virtual phantom (VP1–VP4) that best satisfy the dose constraints. The isodose lines are color‐coded as follows: blue = 200%, orange = 150%, red = 100%, and green = 50% of the prescribed dose. The dotted lines indicate the anatomical contours, with yellow representing the bowel bag, green the sigmoid colon, brown the rectum, blue the bladder, and pink the highrisk clinical target volume.

The results of the MIPS are shown in Figure 4. Parameters with the suffix “S” are associated with SIPS, whereas those with the suffix “M” are associated with MIPS. The dose constraint compliance rates for each virtual phantom were as follows: 64.3% for VP1, 69.6% for VP2, 35.4% for VP3, and 70.8% for VP4. As these rates were relatively low, the spatial relationship between the CTV_HR_ and OARs was adjusted. Following these adjustments, the OAR dose constraint compliance rates improved to 96.4% for VP1, 100% for VP2, 83.3% for VP3, and 87.5% for VP4. Figure 3 displays reference dose distributions that best satisfied the constraints for each virtual phantom.

To facilitate the interpretation of the IPE and IIE across studies, Table 4 summarizes the CVs and statistical comparisons between the IPE and IIE. In addition, in Figure 4, the parameters of SIPS correspond to IPE, while those of MIPS correspond to IIE. Statistically significant differences in CVs are denoted as *: *p*‐value < 0.050, **: *p*‐value ≪ 0.050. In VP1, significant differences in CVs between the SIPS and MIPS were found for the CTV_HR_ V_200%_ value (*p* = 0.001) and for the D_2cc_ values of the bladder, rectum, and sigmoid colon (*p* ≪ 0.05). In VP2, all DVPs exhibited significant differences (CTV_HR_ V_100%_: *p* = 0.001; rectum D_2cc_: *p* = 0.045; bowel D_2cc_: *p* = 0.037; others: *p* ≪ 0.05). In VP3, all CTV_HR_ DVPs differed significantly (all *p* ≪ 0.05), similar to the D_2cc_ values for the bladder (*p* ≪ 0.05) and sigmoid colon (*p* = 0.007). In VP4, significant differences were observed for CTV_HR_ V_100%_ (*p* = 0.016) and for the D_2cc_ values of the bladder and rectum (*p* ≪ 0.05).

**TABLE 4:** Statistical comparison of the coefficients of variation calculated from dose‒volume parameters in the inter‐planner error (IPE) and inter‐institutional error (IIE). An F‐test was conducted to evaluate the statistical significance of the difference between IPE and IIE.

		CTV_HR_				Rectum	Bladder	Sigmoid	Bowel
		D_98%_	D_90%_	V_100%_	V_200%_	D_2cm3_			
VP1	IPE CV [%]	4.69	3.95	0.78	8.77	8.23	8.56	15.05	22.95
	IIE CV [%]	3.40	4.72	0.75	22.42	21.37	26.24	44.40	29.27
	p‐value	0.942	0.277	0.124	<0.001	<0.001	<0.001	<0.001	0.19
VP2	IPE CV [%]	5.63	3.52	2.07	20.99	20.18	8.36	3.30	11.53
	IIE CV [%]	16.03	10.95	4.26	45.81	29.78	42.15	13.86	17.39
	p‐value	<0.001	<0.001	0.001	<0.001	0.046	<0.001	<0.001	0.037
VP3	IPE CV [%]	7.83	7.93	0.38	1.87	10.00	6.12	14.07	23.77
	IIE CV [%]	25.79	17.93	7.84	21.26	11.83	42.36	28.42	29.62
	p‐value	<0.001	<0.001	<0.001	<0.001	0.167	<0.001	0.008	0.104
VP4	IPE CV [%]	9.96	8.10	2.33	13.93	9.53	7.31	18.92	7.71
	IIE CV [%]	6.02	5.67	1.45	18.09	12.38	13.22	21.90	9.86
	p‐value	0.116	0.363	0.016	0.154	0.009	<0.001	<0.001	0.04

An F‐test was conducted to evaluate the statistical significance of the difference between IPE and IIE. Pvalues below 0.001 were reported as <0.001.

## 4 DISCUSSION

HDR‐BT continues to serve as a cornerstone in the management of gynecologic cancers. As highlighted by Han et al., it plays a critical role in treating locally advanced cervical cancer.^38^ In the context of HDR‐BT, accurate applicator reconstruction is essential for achieving an appropriate dose distribution.^39^, ^40^, ^41^, ^42^ Consequently, HDR‐BT planners must be capable of generating robust, evidence‐based treatment plans with both efficiency and precision. To address this need, we developed virtual phantoms to support personnel training, enhance planning consistency, and minimize interobserver variability.

Overall, virtual phantoms were developed for four applicators: Fletcher, Geneva (with three ProGuide sharp needles), VCMC (with three ProGuide round needles), and Venezia (with ProGuide sharp needles) (Figure 1). Dose constraints were defined based on an SIPS (Table 3) and evaluated in an MIPS. The compliance rates for each phantom were as follows: VP1, 64.3%; VP2, 69.6%; VP3, 35.4%; and VP4, 70.8%, indicating that treatment planning remained challenging. This difficulty likely arose from using average DVPs derived from the SIPS as constraints, which created trade‐offs between meeting the dose targets for the CTV_HR_ and OARs. Notably, as the primary goal of a virtual phantom is to support planning practice, overly stringent constraints are not necessary. However, defining new constraints may not resolve these issues, as similar challenges could still arise. Therefore, the dose constraints detailed in Table 3 were retained, and the spatial relationships between the CTV_HR_ and OARs were instead adjusted. The plan that best satisfied the CTV_HR_ dose constraints and achieved the highest D_90%_ and D_98%_ values was identified through the MIPS. Based on this plan, the contour relationships were modified to improve compliance with dose constraints, and the adjusted virtual phantom was designated as the final version. As the virtual phantoms were optimized through MIPS, the suboptimal CTV_HR_ coverage observed in some phantoms was improved, and adherence to OAR constraints became more achievable, thereby providing more effective support for treatment planning practice. Figure 3 presents the dose distribution from the plan that most successfully met the dose constraints, serving as a reference for training purposes.

In the SIPS, the CVs for the CTV_HR_, D_98%_, D_90%_, and V_100%_ were all below 10%, consistent with previous findings^40^ and suggesting an acceptable IPE. However, the CV for V_200%_—the hyper‐dose sleeve—was considerably higher, reaching approximately 20% in the SIPS and exceeding 40% in the MIPS. This suggests that V_200%_ is more sensitive to planner variability than the other DVPs (Table 4 and Figure 3). Despite its clinical relevance^43^, ^44^, ^45^ to certain clinical practices, such as intentionally placing high‑dose regions (e.g., V_200%_) near the applicator or needle body to avoid complications to mucosal tissues, few studies have investigated the variability of V_200%_ in detail. We primarily attribute the large CV of V_200%_ in MIPS to differences in dwell step size and dwell time, which stem from variations in treatment planning policies across planners or institutions. Given the substantial uncertainty observed, efforts to reduce its CV may have clinical significance. For the OARs, the maximum variation within 2SD was approximately 0.8 Gy, with most values falling below 0.5 Gy, consistent with the findings of Wills et al.^40^ These results suggest that, even when a common planning strategy is used, individual planners may produce slightly different dose distributions. In institutions with multiple HDR‐BT planners, establishing clear protocols and ensuring consistent dissemination of planning policies may help reduce such discrepancies and encourage consensus‐based outcomes. In practical applications such as reducing IPE within institutions or training novice planners, the 2SD and CV values reported in this study may serve as useful benchmarks. Training programs aimed at achieving variability below these thresholds could enhance planning consistency and educational outcomes. In clinical practice, despite the differences among planners, the physician ultimately reviews and adjusts the plan based on the implant placement status, the patient's medical history, and the results of prior fractionated radiation therapy. As physicians may intentionally modify the coverage, a certain degree of variation is considered as acceptable.

When comparing IPE and IIE, the latter consistently exhibited greater variability across all virtual phantoms (Tables 2 and 4; Figure 4). For certain DVPs, the CV exceeded 40%, indicating differences in applicator interpretation, planning strategies, and planner experience across institutions. Although some CVs were affected by outliers, the overall trend underscores the importance of addressing the IIE. While earlier studies have investigated IPE within single institutions or examined the effects of deliberately introduced planning errors,^37^, ^38^, ^39^, ^40^ few have evaluated variability across multiple institutions. This study is notable for identifying substantial IIE, thereby highlighting the need for broader standardization. However, a more comprehensive evaluation of IIE would require a larger‐scale MIPS. Reducing the disparity between MIPS and SIPS outcomes is critical for promoting consistency and equity in the quality of HDR‐BT across institutions. Collaborative workshops incorporating virtual phantoms or clinical datasets may help unify planning strategies and treatment protocols, thereby reducing institutional discrepancies and advancing standardized care delivery.

Wilby et al.^9^ reviewed over 100 HDR‐BT phantoms, most of which are physical and inaccessible for routine clinical use. While many are designed for QA/QC applications, few are intended to support treatment planning practice or reduce IPE. Only one phantom has been specifically developed for the training and reduction of interobserver error, and it was tailored for prostate HDR‐BT.^1^ This is unexpected given the well‐established role of HDR‐BT in the treatment of gynecologic cancers.^36^ The lack of accessible training tools for gynecologic HDR‐BT suggests that previous efforts have prioritized physical models, paying limited attention to virtual phantoms, which are digital tools that can be easily shared and adapted across institutions.

The virtual phantom developed in this study aims to bridge this gap by providing a scalable and practical resource for planner training and error reduction. Its digital format enhances usability and accessibility, making it particularly valuable for training purposes and for promoting consistency in treatment planning across diverse clinical environments. We believe that this project constitutes a meaningful contribution to both clinical practice and medical physics education.

Several limitations of this study should be acknowledged. First, the contour shapes used in the phantom did not fully represent actual clinical anatomy. For institutions aiming to provide more clinically realistic training in treatment planning, real patient datasets may offer a more appropriate alternative. This study did not evaluate point dose metrics such as Point A, Point B, or the ICRU reference points, despite their ongoing importance as clinical indicators. This exclusion was based on the widespread adoption of dose volume histogram (DVH)‐based dose evaluation in most institutions. Furthermore, the virtual phantom was specifically designed for use with Elekta applicators and treatment planning systems. As other platforms may involve different operational workflows and planning considerations, the findings reported here may not be directly applicable across all systems. Finally, the primary objective of this study was validating the dose constraints determined through the SIPS. Therefore, the collected DVH parameters were restricted by the small number of institutions participating in the MIPS. The relatively small sample size prevented a more detailed assessment of interobserver variability. Maintaining the relevance of virtual phantoms in HDR brachytherapy presents several practical challenges. Given the wide variety of applicators developed by different manufacturers, achieving broad compatibility demands substantial collaboration, technical coordination, and sustained engagement with industry stakeholders. Furthermore, the rapid evolution of applicator designs—including the introduction of new models and the retirement of older ones—underscores the need for continual updates. Accordingly, efforts toward developing virtual phantoms, including but not limited to our proposed approach, should continue to be pursued as ongoing initiatives. Regular updates will be necessary to ensure continued alignment with contemporary clinical tools and workflows. Such efforts will be critical to maintaining the phantom's long‐term utility, both as a training resource and as a platform for fostering consistency in treatment planning across institutions.

## 5 CONCLUSIONS

We developed a virtual phantom for HDR‐BT treatment planning in cervical cancer management, intended to support planner training, evaluate interobserver error, and potentially serve QA/QC functions. By incorporating multiple applicator types, albeit restricted to four, together with validated dose constraints, the phantom offers a standardized and accessible platform for both educational and clinical use. Its digital format facilitates broad dissemination and adaptability, making it particularly valuable for institutions with limited access to physical phantoms. Continued refinement and periodic updates will be essential to preserve its relevance as a long‐term resource for training and quality assurance in HDR brachytherapy.

## AUTHOR CONTRIBUTIONS

Concept and design: Kotaro I. Data acquisition: Kotaro I., Hiroyuki O., Takahiro U., Yoshihiro U., Yoshifumi O., Yasushi O., Jun T., Jun‐ichi F., Masahiko T. Data analysis: Kotaro I., Naoya M., Hiroyuki O. Drafting the article: Kotaro I., Hiroyuki O., Tatuya O. All authors reviewed and approved the final version.

## CONFLICT OF INTEREST STATEMENT

This study did not require an Institutional Review Board (IRB) approval and written consent because this study does not include any investigations or experiments with human participants or animals performed by any of the authors.

## Data Availability

The data that support the findings of this study are available from the corresponding author upon reasonable request.
